# JNK inhibitor SP600125 protects against lipopolysaccharide-induced acute lung injury via upregulation of claudin-4

**DOI:** 10.3892/etm.2014.1684

**Published:** 2014-04-14

**Authors:** YUELIANG ZHENG, MEIQI ZHANG, YIMING ZHAO, JIE CHEN, BING LI, WENWEI CAI

**Affiliations:** Department of Emergency, Zhejiang Provincial People’s Hospital, Hangzhou, Zhejiang 310014, P.R. China

**Keywords:** JNK inhibitor, acute lung injury, lipopolysaccharide, claudin-4

## Abstract

Although *in vitro* studies have previously demonstrated that mitogen-activated protein kinases are important for the activation of transcription factors and the regulation of proinflammatory mediators, the function of c-Jun NH_2_-terminal kinase (JNK) in acute lung injury (ALI) remains to be fully elucidated. The present study aimed to investigate the effect of the JNK selective inhibitor SP600125 on lipopolysaccharide (LPS)-induced ALI. Pulmonary edema, the expression of inflammatory cytokines and pathological alterations were found to be significantly attenuated in LPS-induced ALI following treatment with SP600125 *in vivo. In vitro*, it was demonstrated that SP600125 administration significantly improved A549 cell viability in a dose-dependent manner using the Cell Counting kit-8 and the 5-ethynyl-2′-deoxyuridine incorporation assay. Furthermore, flow cytometric analysis demonstrated that the apoptotic rate was significantly reduced in a concentration-dependent manner following SP600125 injection. At the molecular level, SP600125 treatment dose-dependently inhibited JNK activation and upregulated claudin-4 expression *in vivo* and *in vitro*. In combination, the results from the present study indicated that the JNK inhibitor SP600125 protected against LPS-induced ALI *in vivo* and *in vitro,* possibly by upregulating the expression of claudin-4.

## Introduction

Acute lung injury (ALI) is a life-threatening clinical disease characterized by acute respiratory distress syndrome, which may be caused by several different factors, including trauma, sepsis and pneumonia (1,2). Despite significant advances in antimicrobial supportive care and therapy to improve the survival rate of patients with ALI, the mortality rate remains high (~40%) (3). Therefore, the development of novel and effective measures against ALI is required.

Several studies have demonstrated that numerous proinflammatory cytokines, including interleukin-6 (IL-6) and tumor necrosis factor-α (TNF-α), are important in the initiation and amplification of lung injury (4). Clinical studies have also demonstrated that elevated levels of proinflammatory cytokines in the serum of patients with ALI are associated with an increased mortality rate (5). Lung-protective ventilation and corticosteroids have been shown to downregulate the level of inflammatory cytokines as well as decrease the mortality rate of patients with ALI (6). Furthermore, previous studies demonstrated that signaling pathways, including the nuclear factor-κB (NF-κB), mitogen-activated protein kinase (MAPK) and phosphatidylinositol 3-kinase pathways, were upregulated in animal models of ALI (7–9). The c-Jun NH_2_-terminal kinase (JNK) is a member of the MAPK family, which has been implicated in the regulation of inflammatory signals and other extracellular signals to intracellular target molecules (10,11). JNK has been identified as a stress-activated protein kinase that phosphorylates c-Jun at two sites on the NH_2_-terminal domain. Inhibition of the JNK signaling pathway leads to the inactivation of transcription factors and other regulatory cellular proteins (12,13). SP600125 is a small molecule that acts as a reversible, ATP-competitive inhibitor of JNK1/2 (14). Due to the effectiveness and specificity of SP600125 in cells and animals experiments, it has been widely used as a pharmacological inhibitor for assessing the role of JNK in the regulation of biological processes (15). In the present study, the therapeutic effect and associated mechanism of SP600125 was analyzed in lipopolysaccharide (LPS)-induced ALI *in vivo* and *in vitro*.

## Materials and methods

### Model establishment

A total of 40 male Wistar rats were randomly divided into four groups (n=10): the control group, LPS group, normal saline group (NS) and the SP600125 group. ALI was induced via intratracheal injection of LPS (Sigma, St. Louis, MO, USA) as previously described (8). Briefly, the rats were anesthetized with pentobarbital sodium followed by intratracheal injection of 5 mg/kg LPS. The rats were then placed in a vertical position and rotated for 1 min to distribute the LPS in the lungs. Normal saline or SP600125 was administered via intraperitoneal injection (15 mg/kg) 10 min after the LPS injection. All experiments were approved by the institutional animal care and research committee of Zhejiang Provincial People’s Hospital (Hangzhou, China).

### Histological examination

The rats were anesthetized 24 h after injury and sacrificed transcardially with saline, followed by treatment with 4% paraformaldehyde. The lungs were immediately removed and post-fixed in 4% paraformaldehyde for 24 h. Paraffin-embedded sections (3 mm thick) were stained with hematoxylin and eosin (H&E) for visualization under a light microscope (magnification, ×200; Leica Microsystems, Wetzlar, Germany).

### Enzyme-linked immunosorbent assay (ELISA)

The levels of claudin-4, TNF-α and IL-6 in the lung tissue samples were measured using an ELISA according to the manufacturer’s instructions (R&D Systems, Minneapolis, MN, USA). The absorbance was measured at 450 nm using a microplate assay (FluoDia T70, Photon Technology International, Lawrenceville, NJ, USA).

### Lung wet to dry (W/D) weight ratio

The severity of pulmonary edema was assessed using the W/D ratio. The left lower lungs were weighed and then dehydrated at 60°C for 72 h in an oven.

### Cell culture and treatments

Human type II-like alveolar epithelial cells (A549) were cultured in RPMI-1640 medium supplemented with 10% fetal bovine serum, 2 mmol/l glutamine, 100 U/ml penicillin and 100 mg/ml streptomycin, and maintained in a humid environment at 37°C and 5% CO_2_. The cells were then treated with LPS (10 μg/ml) and different concentrations of SP600125 (10, 20 and 40 nM). Following 24 h, the cells were collected for further analysis.

### Cell viability assay

Cell viability was evaluated using the Cell Counting kit (CCK)-8 assay (Sigma). In brief, the cells were seeded into 96-well plates at a density of 3×10^3^ cells/well and left to adhere overnight. The cells were then incubated with or without 0–40 nM SP600125. Then, 100 μl CCK-8 was added and incubated in the dark at 37°C for 3 h. The absorbance was determined using the MRX II microplate reader (Dynex, Chantilly, VA, USA) at a wavelength of 450 nm.

### 5-Ethynyl-2′-deoxyuridine (EdU) incorporation assay

A549 cells were exposed to EdU (Invitrogen Life Technologies, Carlsbad, CA, USA) for 2 h at 37°C. The cells were fixed with 4% formaldehyde for 15 min and then treated with 0.5% Triton X-100 for 20 min at room temperature. Following washing with phosphate-buffered saline (PBS) three times, the cells of each well were treated with 100 μl 1 X Apollo reaction cocktail for 30 min. Subsequently, the DNA contents of the cells in each well were stained with 100 μl of Hoechst 33342 (5 μg/ml) for 30 min and visualized using a fluorescent microscope (Leica Microsystems).

### Quantitative polymerase chain reaction (qPCR)

Total RNA was extracted using a TRIzol^®^ kit (Invitrogen Life Technologies) and converted to cDNA using the cDNA Synthesis kit (Takara, Otsu, Shiga, Japan). qPCR was performed using the SYBR-Green Supermix (Invitrogen Life Technologies). The primer sequences used for the PCR reactions were as follows: claudin-4, forward 5′-ACGAGACCGTCAAGGCCAAG-3′ and reverse 5′-GTCCAGGACACAGGCACCATAA-3′; β-actin, forward 5′-GGAGATTACTGCCCTGGCTCCTA-3′ and reverse 5′-GACTCATCGTACTCCTGCTTGCTG-3′. Amplification was performed at 50°C for 2 min, at 95°C for 2 min, followed by 40 cycles of denaturing at 95°C for 15 sec and annealing at 60°C for 30 sec. All the reactions were performed in triplicate. GAPDH was used as a reference gene.

### Western blot analysis

The total protein (20 μg) was separated in each sample using electrophoresis on a 4–20% sodium dodecyl sulfate-polyacrylamide gel and electroblotted onto polyvinylidene difluoride membranes. The membranes were inhibited in a blocking solution and incubated overnight with primary antibodies, including anti-claudin-4, anti-phospho-JNK, anti-JNK and anti-GAPDH (Cell Signaling Technology, Beverly, MA, USA). The membranes were then incubated with anti-rabbit or anti-mouse secondary antibody conjugated with horseradish peroxidase (Pierce Chromatography Cartridges, Thermo Fisher Scientific, Waltham, MA, USA). Immunoreactive bands were detected using the enhanced chemiluminescence kit for western blotting detection by using a ChemiGenius bioimaging system (Syngene, Frederick, MD, USA). Band densities for each protein were determined using Image-Pro Plus 6.0 software (Media Cybernetics, Inc., Bethesda, MD, USA) with GAPDH as a control.

### Flow cytometric analysis

Cell apoptosis was determined using flow cytometry. Briefly, A549 cells were washed with PBS, detached with trypsin and then harvested. The cells were resuspended in 1 ml Hoechst 33258 for 5 min and then washed three times with PBS. Cell apoptosis was detected using the Annexin V-fluorescein isothiocyanate cell Apoptosis Detection kit according to the manufacturer’s instructions (BD Biosciences, Franklin Lakes, NJ, USA).

### Statistical analysis

The data are presented as the mean ± standard deviation and were analyzed using the SPSS statistical software program (SPSS, Inc., Chicago, IL, USA). Comparison between groups was performed using analysis of variance and P<0.05 was considered to indicate a statistically significant difference.

## Results

### SP600125 attenuates LPS-induced ALI in rats in vivo

**Figure 1:**
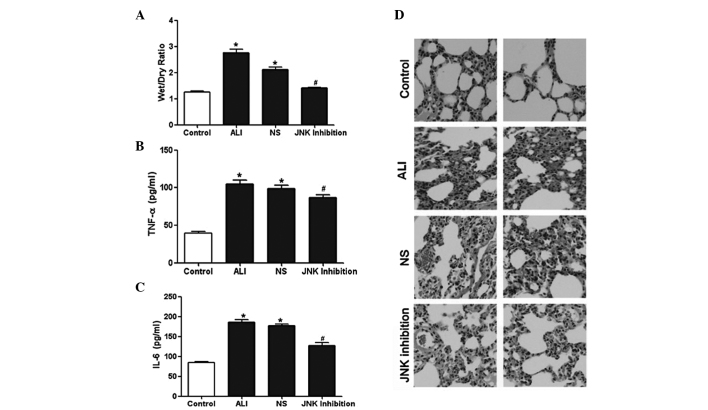
SP600125 attenuates LPS-induced ALI in rats *in vivo*. (A) Following LPS injection with or without SP600125 treatment, the rats were sacrificed and their left lower lungs were obtained in order to determine the wet/dry weight ratio. ^*^P<0.05 vs. control; ^#^P<0.05 vs. ALI. (B and C) An enzyme-linked immunosorbent assay was performed to determine the expression of TNF-α and IL-6 in the bronchoalveolar lavage fluid in the rats from each group. ^*^P<0.05 vs. control; ^#^P<0.05 vs. ALI. (D) Lung tissue sections were stained using hematoxylin and eosin to determine the pathological alterations with or without SP600125 treatment. Representative images are shown from each group (magnification, ×200). Column 1 and 2 show different views in the same group. ALI, acute lung injury; NS, normal saline; JNK, c-Jun NH2-terminal kinase; IL-6, interleukin-6; TNF-α, tumor necrosis factor-α; LPS, lipopolysaccharide.

The lung W/D ratio was analyzed to evaluate pulmonary edema. The LPS-treated rats had higher W/D ratios compared with the control rats. However, the W/D ratio was significantly decreased following administration of SP600125 (Fig. 1A). Furthermore, the results from the ELISA demonstrated that the expression of TNF-α and IL-6 in the bronchoalveolar lavage fluid (BALF) in the LPS-treated rats was markedly increased compared with the rats in the control group. However, the expression levels of TNF-α and IL-6 in the BALF in rats in the SP600125 group were significantly decreased (Fig. 1B and C). To assess the pathological alterations, H&E staining was performed and the results revealed evidence of infiltration of inflammatory cells, interstitial edema and interalveolar septal thickening, as well as intra-alveolar and interstitial hemorrhage. However, following treatment with SP600125, the pathological changes in the lung tissues of the rats markedly decreased (Fig. 1D). These results demonstrated that SP600125 treatment alleviated LPS-induced ALI *in vivo.*

### Effect of SP600125 administration on A549 cell viability and apoptosis

**Figure 2:**
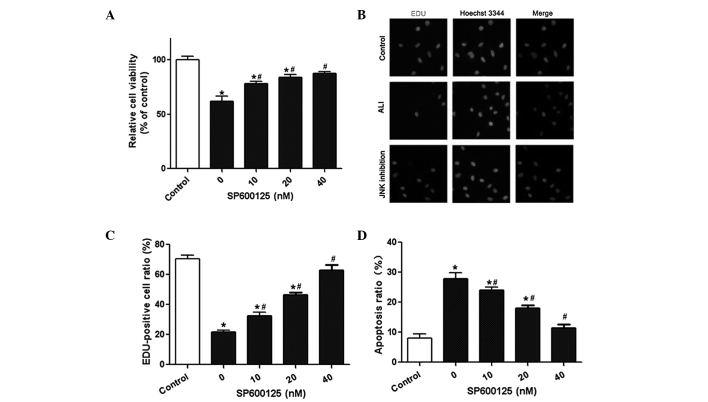
Effect of SP600125 administration on A549 cell viability and apoptosis. (A–C) Cells were treated with different concentrations of SP600125 (between 0 and 40 nM). The Cell Counting kit (CCK)-8 and EdU incorporation assay were then performed to measure the A549 cell viability. ^*^P<0.05 vs. control; ^#^P<0.05 vs. ALI. (D) A549 cells were treated with SP600125 and the apoptotic rate was detected using flow cytometry. ^*^P<0.05 vs. control; ^#^P<0.05 vs. ALI. ALI, acute lung injury; NS, normal saline; JNK, c-Jun NH2-terminal kinase; EdU, 5-ethynyl-2′-deoxyuridine.

The lung epithelial cell line A549 was used in the present study to investigate the effect of SP600125 on A549 cell viability and apoptosis. The results from the CCK-8 assay revealed that cell viability was significantly reduced following LPS treatment. By contrast, SP600125 administration significantly improved the viability of A549 cells in a dose-dependent manner (Fig. 2A). The results from the EdU assay were consistent with these results (Fig. 2B and C). Flow cytometry was used to determine the effect of SP600125 on cell apoptosis. The results demonstrated that SP600125 treatment significantly reduced the apoptotic rate of A549 cells in a dose-dependent manner (Fig. 2D). In combination, these results suggested that SP600125 was able to protect against LPS-induced ALI in rats *in vitro*.

### Effect of SP600125 on claudin-4 expression and JNK phosphorylation in vivo and in vitro

**Figure 3:**
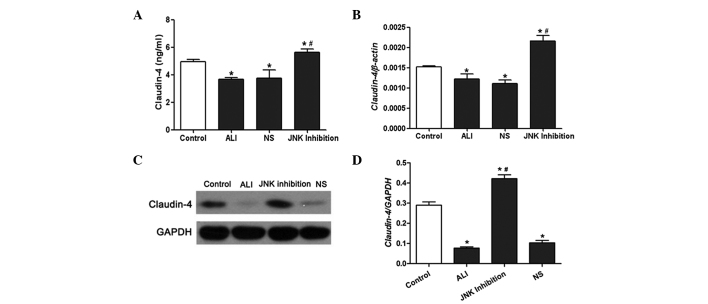
SP600125 treatment increases the expression of claudin-4 in BALF and lung tissues in the rats in each group. (A) Following LPS injection with or without SP600125 treatment, the cells in the BALF were collected and an enzyme-linked immunosorbent assay was used to measure the expression levels of claudin-4. ^*^P<0.05 vs. control; ^#^P<0.05 vs. ALI. (B) mRNA expression of claudin-4 in the lung tissues was determined using quantitative polymerase chain reaction with β-actin as a control. ^*^P<0.05 vs. control; ^#^P<0.05 vs. ALI. (C and D) Claudin-4 protein expression in the lung tissues was analyzed by western blotting using anti-claudin-4 and anti-GAPDH antibodies. The expression of each protein was measured using the relative band intensities. ^*^P<0.05 vs. control; ^#^P<0.05 vs. ALI. ALI, acute lung injury; NS, normal saline; JNK, c-Jun NH2-terminal kinase; BALF, bronchoalveolar lavage fluid; LPS, lipopolysaccharide.

**Figure 4:**
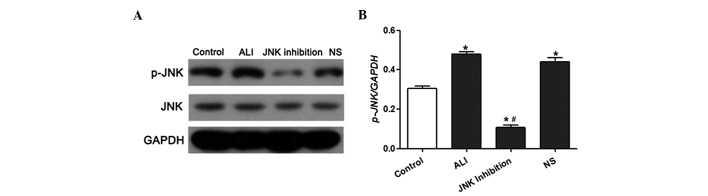
Effect of SP600125 on claudin-4 expression and JNK phosphorylation *in vivo*. (A and B) Rats were sacrificed following LPS injection with or without SP600125 treatment and the total protein from the lung homogenates was obtained and analyzed in order to determine p-JNK phosphorylation using western blotting with anti-phospho-JNK, anti-JNK and anti-GAPDH antibodies. The expression of each protein was measured using the relative band intensities. ^*^P<0.05 vs. control; ^#^P<0.05 vs. ALI. ALI, acute lung injury; NS, normal saline; p-JNK, phosphorylated c-Jun NH2-terminal kinase; LPS, lipopolysaccharide.

The results from the ELISA demonstrated that the expression of claudin-4 in BALF in the LPS-treated rats was markedly lower compared with the rats in the control group. However, the expression levels of claudin-4 in BALF in the rats from the SP600125 group were significantly increased (Fig. 3A). The mRNA and protein expression of claudin-4 was significantly reduced in rat lung tissues treated with LPS; however, this was reversed following administration of SP600125 (Fig. 3B–D). Western blot analysis demonstrated that JNK phosphorylation in lung tissues was significantly increased following LPS treatment. However, SP600125 administration led to a reduction in JNK phosphorylation in the lung tissues of LPS-induced ALI rats (Fig. 4A and B).

**Figure 5:**
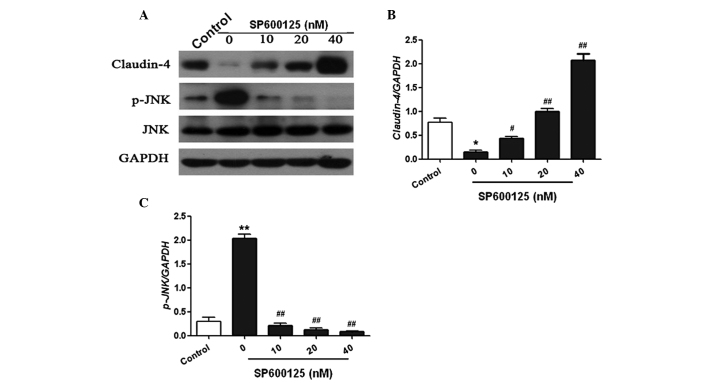
Effect of SP600125 *in vitro* on the expression of claudin-4 and JNK phosphorylation. The cells were treated with different concentrations of SP600125. The cells were then lysed and the protein was extracted in order to determine the expression of (A and B) claudin-4 and (C) p-JNK phosphorylation by western blot analysis using anti-claudin-4, anti-phospho-JNK, anti-JNK and anti-GAPDH antibodies. The expression of each protein was measured using the relative band intensities. ^*^P<0.05 vs. control; ^#^P<0.05 vs. ALI. ALI, acute lung injury; NS, normal saline; p-JNK, phosphorylated c-Jun NH2-terminal kinase.

Furthermore, the *in vitro* study demonstrated that the expression of claudin-4 was markedly reduced following LPS injection in A549 cells. However, treatment with SP600125 significantly increased the expression of claudin-4 in a dose-dependent manner (Fig. 5A and B). In addition, JNK phosphorylation was significantly increased following LPS treatment. However, SP600125 treatment dose-dependently reduced JNK phosphorylation in A549 cells (Fig. 5C). These results demonstrated that SP600125 inhibited JNK activation and upregulated claudin-4 expression levels *in vivo* and *in vitro*.

## Discussion

ALI is an acute and progressive respiratory disease, which is characterized by progressive diffuse bilateral pulmonary edema and inflammation (16). ALI has a high mortality rate and 40–50% of patients succumb to multiple organ failure (3). In the present study, the effect of SP600125, a JNK selective inhibitor, in LPS-induced ALI and its underlying mechanism was investigated.

A previous study demonstrated that, LPS, located on the outer membrane of Gram-negative bacteria, acted as a pro-inflammatory reaction factor in infectious diseases and resulted in inflammatory reactions *in vivo* (17). Pulmonary edema, a typical pathological change observed in ALI, was found to reduce lung compliance and decrease pulmonary gas exchange (18). The present study found that SP600125 significantly attenuated LPS-induced pulmonary edema *in vivo,* as shown by the lung W/D ratio. Numerous studies have demonstrated that various inflammatory mediators are involved in the pathogenesis of ALI. Among them, TNF-α and IL-6 are considered to be the most important inflammatory mediators in the innate immune response (4). According to the results from the present study, SP600125 treatment markedly decreased the expression of TNF-α and IL-6 in BALF induced by LPS stimulation. In addition, the results demonstrated that typical ALI pathological alterations were reduced by SP600125 administration. These results indicated that SP600125 effectively inhibited LPS-induced pulmonary edema and inflammation *in vivo*.

Next, the effect of SP69600125 in cultured cells was investigated. The lung epithelial cell line A549 was used in the present study to investigate the effect of SP600125 on A549 cell viability and apoptosis. The results demonstrated that SP600125 significantly improved A549 cell viability in a dose-dependent manner using the CCK-8 and EdU incorporation assays. Apoptosis is the process of programmed cell death that is important in cell growth and maintaining cellular homeostasis, and is regulated by numerous signaling pathways (19,20). The present study demonstrated, using flow cytometry, that SP600125 treatment dose dependently reduced the apoptotic rate of A549 cells. The results from the present study demonstrated *in vitro* that SP600125 administration promoted cell viability and reduced cell apoptosis, therefore protecting against LPS-induced lung injury.

In addition, the present study investigated the molecular mechanism underlying the protective effect of SP600125 on LPS-induced ALI. JNK is known to be an important upstream regulator of the induced expression of inflammatory mediators in response to stress, cytokines and cytoskeletal reorganization (21,22). Previous studies have demonstrated that JNK inhibition by SP600125 suppressed the release of TNF-α into BALF, as well as inhibited the expression of matrix metalloproteinases in synoviocytes and in inflammatory arthritis (23,24). Another study revealed that the inhibition of JNK suppressed LPS-induced increases in the DNA binding activity of NF-κB by downregulation of the phosphorylation of inhibitor κB-α (25). The results from the present study demonstrated that SP600125 significantly inhibited the JNK signaling pathway *in vivo* and *in vitro*. Claudins are a family of proteins that are important components of tight junctions, where they establish the paracellular barrier controlling the molecular flow between the cells of an epithelium (26). Claudin-4, one member of the claudin family, has been previously reported to be required for maximal epithelial barrier function, including alveolar fluid clearance in mice (27,28). The levels of claudin-4 affect paracellular transport by conferring relative chloride selectivity and decreasing ion conductance, suggesting that claudin-4 is important in determining alveolar fluid clearance rates in human lungs (29). In the present study, claudin-4 expression was found to be significantly increased following SP600125 administration *in vivo* and *in vitro*, suggesting that the protective effect of SP600125 was partly mediated by claudin-4.

In conclusion, the present study demonstrated for the first time, to the best of our knowledge, that the JNK inhibitor SP600125 protected against LPS-induced ALI *in vivo* and *in vitro,* possibly by upregulating the expression of claudin-4.
